# A model to differentiate WAD patients and people with abnormal pain behaviour based on biomechanical and self-reported tests

**DOI:** 10.1007/s00414-021-02572-5

**Published:** 2021-03-27

**Authors:** Merylin Monaro, Helios De Rosario, José María Baydal-Bertomeu, Marta Bernal-Lafuente, Stefano Masiero, Mónica Macía-Calvo, Francesca Cantele, Giuseppe Sartori

**Affiliations:** 1 Department of General Psychology, University of Padova, via Venezia 8, 35131 Padova, Italy; 2 Instituto de Biomecánica de Valencia, Universitat Politècnica de Valencia, Ed. 9C. Camino de Vera s/n, 46022 Valencia, Spain; 3 CIBER de Bioingeniería, Biomateriales Y Nanomedicina (CIBER-BBN), Zaragoza, Spain; 4 MAZ, Academia General, Mutua Colaboradora con la Seguridad Social nº 11. AvenidaMilitar 74, 50015 Zaragoza, Spain; 5 Department of Neuroscience, Section of Rehabilitation, University of Padova, Via Nicolò Giustiniani, 5, 35128 Padova, Italy

**Keywords:** WAD, Whiplash, Malingering detection, Whiplash kinematic test, Whiplash self-report questionnaire

## Abstract

The prevalence of malingering among individuals presenting whiplash-related symptoms is significant and leads to a huge economic loss due to fraudulent injury claims. Various strategies have been proposed to detect malingering and symptoms exaggeration. However, most of them have been not consistently validated and tested to determine their accuracy in detecting feigned whiplash. This study merges two different approaches to detect whiplash malingering (the mechanical approach and the qualitative analysis of the symptomatology) to obtain a malingering detection model based on a wider range of indices, both biomechanical and self-reported. A sample of 46 malingerers and 59 genuine clinical patients was tested using a kinematic test and a self-report questionnaire asking about the presence of rare and impossible symptoms. The collected measures were used to train and validate a linear discriminant analysis (LDA) classification model. Results showed that malingerers were discriminated from genuine clinical patients based on a greater proportion of rare symptoms vs. possible self-reported symptoms and slower but more repeatable neck motions in the biomechanical test. The fivefold cross-validation of the LDA model yielded an area under the curve (AUC) of 0.84, with a sensitivity of 77.8% and a specificity of 84.7%.

## Introduction

Whiplash-related injuries are estimated to account for approximately 80% of all traffic injuries [1], representing a critical health, social, and economic issue [2]. For instance, whiplash is in Germany is the most common consequence of road traffic accidents, with approximately 20,000 cases yearly and costing insurance companies more than 500 million euro [3]. Although the numbers vary significantly across countries, whiplash-related injuries in Europe were estimated to cost annually up to 10 billion euros [4], with an increment in recent years [1].

Whiplash is characterized by a high variability of symptoms, commonly referred to as whiplash associated disorders (WAD) [5]. They may encompass diffuse neck pain, neck stiffness, back pain and back stiffness, headaches, fatigue, vision disorders, and dizziness. Patients may also report anxiety, depressive symptoms, difficulties in concentration, and memory deficit [6]. Although it is recommended to conduct an in-depth evaluation, collecting a range of circumstantial, clinical, and instrumental data [7], the diagnosis of whiplash is still largely based on self-reported symptoms [8], as the current medical diagnostic techniques are unable to accurately detect soft tissue injuries, which are predominant in minor WAD [9]. This makes whiplash a clinical condition hard to diagnose and, at the same time, easy to simulate. Moreover, the lack of objective evidence of symptoms, together with the prospect of obtaining compensation, may encourage policyholders to feign or exaggerate their symptomatology [1]. In support of this, Cassidy and colleagues (2000) showed that the elimination of financial compensations for pain and suffering was associated with a drop in the number of insurance claims, as well as with a faster recovery [10]. Similarly, in countries where compensation for late whiplash-related injuries is not formally provided (e.g., Lithuania, Greece), patients rarely develop chronic symptoms [3, 10].

Overall, the literature indicates that the prevalence of malingering among individuals presenting late whiplash-related symptoms is significant [11], especially in litigation cases, where a proportion of up to 60% is reached [12]. Therefore, the economic loss linked to fraudulent injury claims is huge, making the detection of exaggerated or feigned whiplash-related symptoms a priority. Consequently, valid and accurate tools that allow practitioners in a medicolegal context to identify malingered WAD are needed.

### WAD malingering detection

One traditional approach to detecting malingering is the qualitative analysis of the symptomatology, applying clinical and epidemiological rules to forensic practice. For example, the discrepancy method consists of qualitatively analysing the reported symptoms considering their incidence in the clinical population affected by the claimed disorder. In short, the plausibility of the reported symptoms profile is evaluated by comparing it with the typical clinical profile [13]. It has been shown that malingerers tend to report a larger number of symptoms compared with the clinical population (indiscriminate symptom endorsement), including rare and impossible symptoms, that is, symptoms that are infrequent or unlikely to be seen among genuine clinical patients [14, 15]. Moreover, malingerers are more prone to amplify the severity of the disorder, describing their symptoms as “extreme” or “unbearable”. In fact, there is a common misconception that reporting more symptoms or overreporting their severity increases the probability of being identified as affected by a genuine syndrome. Moreover, as malingerers do not have in mind a clear representation of the pattern of symptoms typically associated with a specific disease, they can show a symptom, or a pattern of symptoms, even if it is not plausible for the disease they are trying to feign [16, 17].

This evidence has contributed to building tools for the evaluation of malingering, especially in the psychiatric field. For instance, the Structured Inventory of Malingered Symptomatology (SIMS), a self-report questionnaire based on asking about rare and impossible symptoms, was conceived to detect malingering of psychiatric disorders and cognitive impairments [18]. Concerning the simulation of whiplash, tools that check the presence of non-organic signs, namely behavioural signs that are not compatible with the organic injury, have been proposed. Sartori et al. developed the Whiplash Syndrome Questionnaire [19], a self-report measure that includes eight scenarios, each with ten daily life actions (e.g., driving in traffic for 40 min) that responders are asked to rank according to the ease with which they can be performed. The rationale is that only patients with an authentic WAD can recognize easy versus non-easy daily life actions to perform. In a small validation sample, the questionnaire was shown to correctly identify 94% of the simulators and 84% of the exaggerators. Sobel et al. [20] proposed a tool to identify abnormal illness behaviours, which consists of the clinical observation of eight non-organic cervical signs (superficial and nonanatomic tenderness; head/shoulder/trunk rotation; range of motion; sensory loss and motor loss; overreaction). The presence of two or more signs indicates a suspect of simulation. However, the accuracy, sensitivity, and specificity of the use of non-organic signs for WAD malingering detection are not known [21]. Several authors criticized this approach, arguing that these signs are not necessarily indicative of malingering, as they may be a response affected by fear from injury and development of chronic incapacity [22, 23]. On the other hand, some authors support the use of non-organic signs from the Sobel test in clinical practice as a starting point of simulation suspicion from a physical point of view and within a holistic approach to the patient [24, 25].

Other methodologies proposed in the literature to detect malingered WAD are differential spinal blocks implementation, thermographic amytal evaluation, pentothal administration, isometric strength testing [26], posturography technique [27], and mechanical testing [22]. In particular, in the mechanical approach, the kinematic parameters of cervical and neck mobility are used as cues to detect whiplash malingering [28, 29]. The rational is that the evaluation of movements performed multiple times and/or under different circumstances helps reveal inconsistencies between repeated performances or abnormal and improbable patterns of impairment. Similarly, the Fly test records head movements while participants are following a fly, and computes three kinematic parameters (amplitude accuracy, time on target, and jerk index) that differentiate patients with genuine WAD from fakers with an accuracy of 71.8–81.5% [30] identifying abnormal movement patterns in terms of amplitude, time and jerk index.

Finally, completely different strategies derive from the studies of the cognitive mechanisms of deception [31]. The cognitive-based lie detection techniques rely on the evidence that lying is more cognitively demanding than truth telling [32], and this greater cognitive effort is reflected in the time to respond to a stimulus (e.g., a question about whiplash symptoms). Among these, the autobiographical Implicit Association Test (aIAT), which detects liars focusing on response times (RTs) during a classification task, appears particularly promising [33]. Notably, in a preliminary study, the aIAT was successfully applied to detect the malingering of whiplash-related injuries, showing an accuracy of approximately 90% [34]. Other encouraging malingering detection techniques include mouse dynamics [35] and keystroke analysis [36]. Nevertheless, the literature on these techniques is still in its infancy, and further studies are needed to apply and validate them for WAD malingering detection.

### Aim of the study

Practitioners in the medicolegal context are still looking for solid criteria to detect WAD malingering and symptoms exaggeration. As reported above, various strategies have been proposed in previous literature. However, most of them have not been consistently validated and tested to determine their accuracy in detecting feigned whiplash. The aim of the present study was to merge two different approaches from among those most commonly used by forensic practitioners to detect WAD malingering – the mechanical approach and the qualitative analysis of the symptomatology – to obtain a malingering detection model based on a wider range of indices, both biomechanical and self-reported. To this end, we tested malingerers and genuine clinical patients using a kinematic test used in the assessment of WAD-related pain [37], and a self-report questionnaire, which was built ad hoc for this study based on rare and impossible whiplash symptoms. Then, the collected measures were used to train and validate classification models, investigating the accuracy, sensitivity, and specificity of the two approaches together in detecting WAD malingerers.

## Methods

### Participants

A cohort of subjects was measured between November 2018 and February 2020, in Italy (Unit of Rehabilitation, University-General Hospital of Padova, UNIPD) and Spain (Hospital MAZ). All participants gave informed consent to participate in the study and process the data recorded in the tests. The methodology was approved by the Ethics Committee of UNIPD.

The cohort consisted initially of two groups of people: one group of patients with cervical pain due to a traffic accident with whiplash, classified on the I-to-III scale of the Quebec classification [5], and people who had recovered from previous episode with similar characteristics, without current signs of neck or other musculoskeletal pain. Two examiners assessed the subjects belonging to the patient group, looking for cervical nonorganic signs [20], and classified them into a “control” group of patients (C) and “suspects” of abnormal or exaggerated pain behaviour (S). Since such classification based on experience and nonorganic signs may not be directly related to the patient’s intention, and might be affected by personal bias, a third group of participants was constituted by “fakers”, recovered patients who were asked by the researchers to reproduce the symptomatology of their previous painful episode (F), which could be compared to the S group and previous studies with similar participant profiles [28, 29].

### Measurements

All subjects completed a Neck Pain Symptoms Questionnaire (NPSQ). The NPSQ was conceived ad hoc for this study, according to the previous literature about rare and impossible symptoms strategy [18]. It consisted of 65 “true/false” questions asking for possible (n = 21; e.g., *I often suffer of muscle stiffness*), rare (n = 14; e.g., *I lost the sensibility in both upper limbs*), and impossible (n = 30; e.g.; *Sometimes I hear a constant sound in my ears*) whiplash-related symptoms. The questions were identified by investigators of the Instituto de Biomecánica (IBV) based on the most solid clinical literature on WAD, and then translated into Italian and Spanish (an English version of the questionnaire is reported in Annex A Table 5). The NPSQ was administered through an online form.

Neck motion was analysed as described by De Rosario et al. [37], measuring range of motion (ROM), maximum angular velocity (MAV), phase-area ratio (PAR), and harmonicity (HARM) of flexion–extension (FE), rotation (R), and lateral flexion (LF) movements. Spanish patients were measured using the NedCervical/IBV system, whereas the Italian cohort was measured using the WAAS/IBV system. The systems differed in the instrumentation (optical sensors in NedCervical/IBV vs. inertial sensors in WAAS/IBV), but they implemented the same measurement protocol, and a suitable placement of sensors and postural calibration were considered to ensure that the discrepancies between instruments remained below 3 degrees for ROM, 2% of the range of MAV, and less than 1% of PAR and HARM [37].

### Statistical analysis

The agreement of the examiners’ judgements was tested using Fleiss’ kappa [38], and the characteristics of the subjects (sex and age) were compared across sites and groups to verify that the sample was balanced (Chi-squared test of homogeneity for sex, and ANOVA for age). Then, the distributions of the NPSQ scores and the normalized biomechanical parameters were compared across the three patient groups, using an ANOVA and post-hoc comparisons, to investigate what variables might be the most promising discriminators between patients who are not suspect of malingering pain at all (C) and the rest (S or F), or between “suspects” (S) and healthy people deliberately feigning pain (F). Pearson’s correlation coefficients were also calculated between the measured variables to spot potential multicollinearities that should be avoided in the subsequent steps [39].

A minimal set of weakly correlated and potentially discriminant variables was chosen according to those results to conduct a linear discriminant analysis (LDA). The study sample was split into five subsets, which were used to test the goodness of the model to distinguish binarily between non-suspect patients (C) and the other groups, in a fivefold cross-validation.

All analyses were conducted using the R package for statistical computing [40–44].

## Results

### Description of the participant sample

One hundred and five people participated in the study, classified into three groups, as shown in Table 1. Of the patients, 26% were assigned to the “suspect” group (S), and a similar number of “fakers” (F) were recruited. The opinions of the two judges who classified the patients showed a good level of agreement (Fleiss’ κ = 0.928). Participants’ ages ranged between 22 and 85 years (average 39.2, std. dev 13.5), and there was a majority of female participants (70%), but there were no significant differences in either sex (*p* > 0.58) or age (*p* > 0.37) between sites or groups.

**Table 1:** Distribution of the participants

Country	C	S	F	Total
Italy	35	9	11	55
Spain	24	12	14	50
Total	59	21	25	105

### Selection of NPSQ parameters

Controls reported fewer symptoms of all types (possible, rare, and impossible) than the other two groups did, but that difference was not significant for the impossible symptoms (Table 2), so it was left out of the model. Furthermore, the total NPSQ score was discarded, because it was strongly correlated with possible, rare, and impossible single scores (*ρ* > 0.77). The NPSQ “possible” and “rare” scores were retained as potentially discriminatory variables, with a moderate correlation between them (*ρ* = 0.54).

**Table 2:** Average (and std. dev) score for each subset of symptoms of the NPSQ in the three groups (C, F, and S), and results of the post-hoc comparisons of differences between groups

Symptoms	C	S	F	C vs. F/S	F vs. S
Possible	14.7 (0.4)	17.2 (0.7)	17.2 (0.7)	*F*_(1,100)_ = 14.08, *p* = 0.000	*F*_(1,100)_ = 0.00, *p* = 0.987
Rare	3.4 (0.3)	4.5 (0.6)	5.1 (0.5)	*F*_(1,100)_ = 7.49, *p* = 0.007	*F*_(1,100)_ = 0.63, *p* = 0.431
Impossible	4.0 (0.5)	5.3 (0.8)	5.3 (0.8)	*F*_(1,100)_ = 3.46, *p* = 0.066	*F*_(1,100)_ = 0.00, *p* = 0.979
Total	22.1 (1.0)	27.0 (1.6)	27.6 (1.5)	*F*_(1,100)_ = 12.26, *p* = 0.001	*F*_(1,100)_ = 0.06, *p* = 0.803

### Selection of biomechanical parameters

All biomechanical parameters were significantly different between controls and the other two groups, which were hardly distinguishable from each other (Table 3), but it was necessary to choose only a minimal set of those parameters, and pick only one movement (FE, R, or LF) for each parameter, because all parameters were strongly correlated between movements (*ρ* between 0.67 and 0.91). Due to the smaller differences observed in HARM compared with the other parameters, and in all parameters comparing LF and the other movements, they were left out of the model. Furthermore, ROM and MAV were strongly correlated in all movements (*ρ* > 0.70), so the choice was narrowed down between a pair of parameters (either ROM or MAV plus PAR) for FE or R. The set that minimized the correlations between variables was MAV and PAR in R (*ρ* = -0.12).

**Table 3:** Statistics of the post-hoc comparisons of differences between groups for the normalized biomechanical parameters: *F*_(1,99)_ (*p*-value)

	**ROM**	**MAV**	**HARM**	**PAR**
**FE**				
C vs. F/S	13.01 (0.000)	13.55 (0.000)	5.51 (0.021)	16.22 (0.000)
F vs. S	2.21 (0.140)	1.86 (0.176)	0.19 (0.662)	0.02 (0.887)
**R**				
C vs. F/S	14.00 (0.000)	15.38 (0.000)	6.13 (0.015)	13.43 (0.000)
F vs. S	0.19 (0.667)	0.46 (0.498)	1.90 (0.171)	0.29 (0.592)
**LF**				
C vs. F/S	7.35 (0.008)	10.53 (0.002)	4.76 (0.031)	8.28 (0.005)
F vs. S	0.10 (0.751)	2.38 (0.126)	0.91 (0.343)	0.72 (0.398)

Note: range of motion (ROM), maximum angular velocity (MAV), harmonicity (HARM), phase-area ratio (PAR), flexion–extension (FE), rotation (R), lateral flexion (LF), healthy people deliberately feigning pain (F), “control” group of patients (C), people “suspects” of abnormal or exaggerated pain behaviour (S).

### Linear discriminant analysis

Considering the previous analysis, the LDA model was fitted using the “possible” and “rare” NPSQ scores, plus MAV(R) and PAR(R) as predictors. The coefficients of the linear discriminant functions (LD1 and LD2, cf. Table 4) indicate that the LD1 increased as more possible symptoms but fewer rare symptoms were reported on the NPSQ, and when PAR increased or MAV decreased. The same relationships were obtained for LD2, but with different proportions: possible symptoms and MAV were more relevant for LD1 and less for LD2 than were rare symptoms and PAR.

**Table 4:** Coefficients of the discriminant functions (LD1, LD2)

	**LD1**	**LD2**
NPSQ possible	0.202	0.18
NPSQ rare	-0.024	-0.454
MAV(R)	-1.086	-0.041
PAR(R)	0.4	0.134

**Fig. 1:**
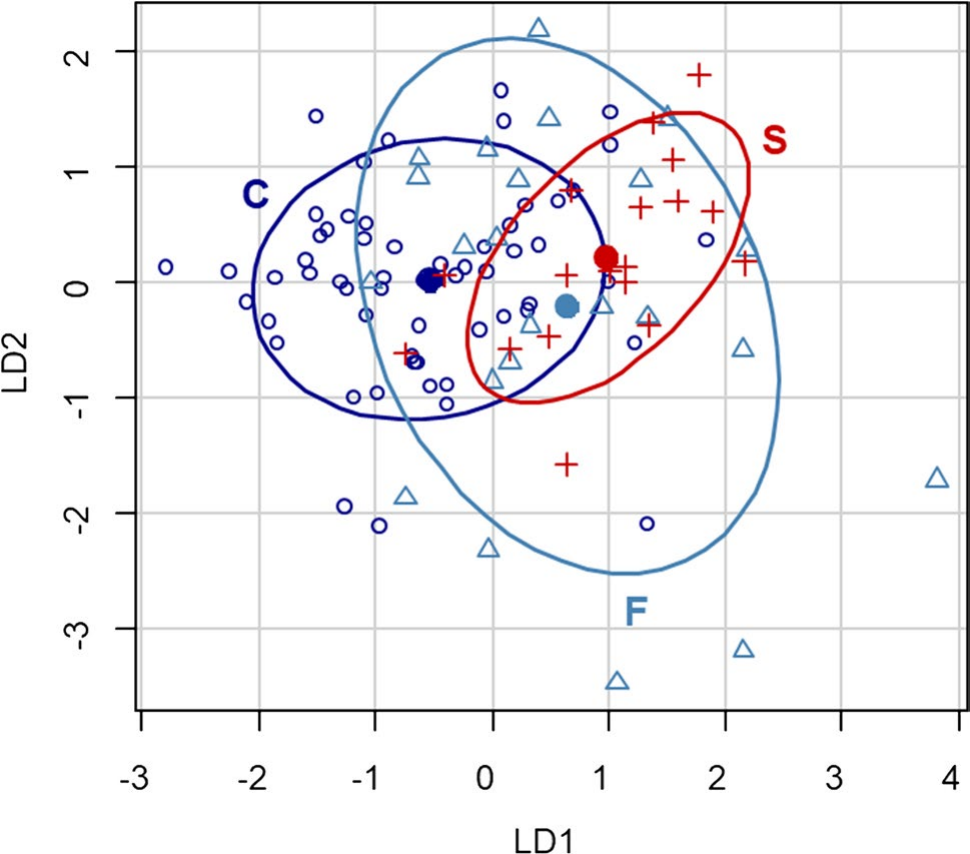
Distribution of the three groups of participants in the space of the discriminant functions: C represented as empty circles, S as crosses, and F as triangles. The ellipses and their centres represent the confidence regions around the group means, within a distance of ± 1 standard deviation from the group means. (Mahalanobis distances, which have a different scale in each axis considering the variance structure of the data.)

Figure 1 shows the distributions of cases in the space of the discriminant functions. The two groups of patients (C and S) were separated, mainly in the direction of LD1. On the other hand there was a large overlap between both groups with F, due to its large dispersion, specially in the axis of LD2.

**Fig. 2:**
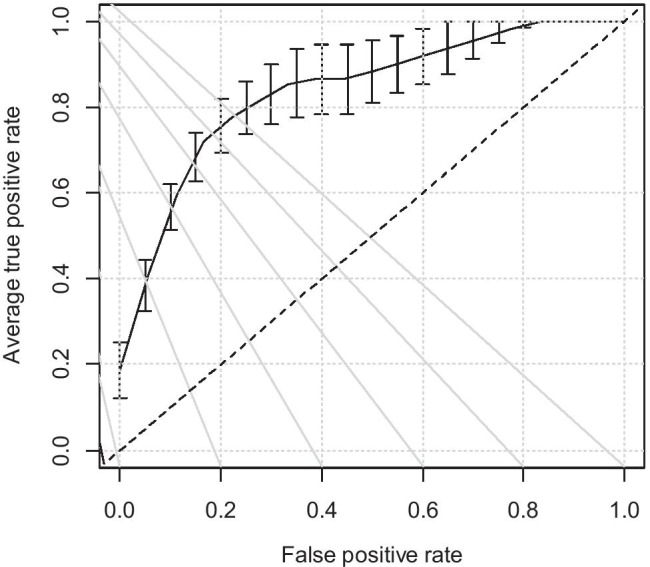
Average ROC curve and standard errors for the fivefold cross-validation

The fivefold cross-validation of the LDA model as a binary classifier between the groups of patients C and S yielded an average area under the curve equal to 0.84 (Fig. 2), which can be considered “excellent discrimination” [39]. At the standard cut-off point ($\mathrm{Pr}\left(C\right)=0.5$), sensitivity was 77.8% (people in the S group successfully classified as “suspects”), and specificity was 84.7% (people in the C group successfully classified as “non-suspects”). On the other hand, the model could not label the subjects of the F group better than a random classifier could.

## Discussion and conclusion

This study looked for signs in self-reported measurements and biomechanical tests that could be used to differentiate ordinary patients suffering WAD and people with abnormal pain behaviour, who might be feigning or exaggerating their symptoms. Our study sample incorporated a group of suspected malingerers among real patients, which were expected to represent better the kind of malingerers that physicians encounter in daily practice, as well as a group of purposeful fakers, less realistic but whose intent could not be misjudged.

The statistical analysis showed that those signs were a larger number of symptoms reported on the NPSQ — particularly a greater proportion of “rare” symptoms vs. “possible” symptoms — and slower but more repeatable neck motions in the biomechanical test (smaller MAV and greater PAR).

The discriminant model built with those variables presented a large overlap between actual patients who were “suspect” of some response bias and former, recovered patients who were asked to fake the symptoms, although the quantity of rare symptoms reported and the repeatability of neck motion in the tests tended to be greater in the “suspect patients”. Such a model had a good discriminant ability when used for a binary classification between the two different types of patients. At the standard cut-off point, the model showed higher specificity than sensitivity to detect possible simulators (i.e. it is a “conservative” model that failed on the safe side from the patient’s perspective).

These results are comparable with those obtained by Baydal-Bertomeu et al. [28] with a similar biomechanical test, although in other, less challenging conditions. The present study was conducted at two sites, with a greater variety of patients, including not only people voluntarily “faking” their behaviour, but also patients who were suspected of performing abnormal or exaggerated pain behaviors; to strengthen the analysis, the test also included movements in different directions, the motion parameters were normalized, and self-reported questionnaires were added. The influence of MAV and PAR in the model was similar in both studies, as well as the sensitivity/specificity balance, although their values were about 10% smaller in the present study.

Concerning the NPSQ, this is one of the first self-reported measures specifically developed to detect whiplash malingering. Indeed, the only instrument already present in the literature is the Whiplash Syndrome Questionnaire [19], for which an accuracy of 90% in detecting WAD malingerers is reported. Although this tool performs better than the questionnaire we presented in this study (NPSQ), it was cross-validated in a sample of 40 exclusively Italian participants.

Finally, regarding the Sobel test, no studies in the literature report metrics about its accuracy in detecting malingering, according to the fact that this test was conceived to detect non-organic signs and not to classify malingerers.

The overlap between the groups of “suspects” and actual “fakers” in the model parameters indicates that the signs that raised the suspicion of the examiners partly coincided with the patterns exhibited by people who were asked to feign. But contrary to our initial hypothesis, the group of “suspects” was more clearly differentiated from the “normal” patients than from the “fakers”. Because the assignment of patients to the “suspect” group was the result of subjective assessment and agreement by two examiners, there might have been a selection bias that narrowed down the profile of that group, such that only those who exhibited unusual traits of exaggeration were singled out as suspects. If that was the case, the values of sensitivity and specificity of the model would be overestimated and underestimated, respectively. Alternatively, it might be that the unrealistic condition of the group of fakers made that group unusually heterogeneous.

Empirical research on the phenomena of sub-maximal effort, insincerity of effort, or even the simulation of pain implicitly entails the difficulty of selecting patients suspected of this behaviour [45]. More generally, this is an experimental issue common to all research in the field of deception detection [36]. The nature of the problem prevents an objective and reliable investigation. This has led some researchers to adopt a strategy whereby insincerity has been modelled by simulation in normal subjects. In our case, we chose to use the Sobel method, although we are fully aware that non-organic signs from the Sobel test do not have to be directly related to the patient’s intention to simulate pain in order to obtain a secondary benefit. Many groups of researchers have attempted to clarify the concept of simulation and its relationship to secondary gain, which could facilitate the selection of groups in this research field [46–49]. However, this information does not seem to be widely agreed upon among clinicians, due, in part, to unresolved theoretical issues.

Another aspect of the study that might have an impact on the results was the selection of the classification tool. LDA was chosen because its simple mathematical model facilitated the interpretation of its coefficients and understand how the results of the tests influenced the classification, and also allowed working with moderately sized samples, and the comparison with previous literature. Other machine learning classifiers, as Support Vector Machines, Neural Networs or K-nearest Neighbour (K-NN), might provide better fits, if trained with properly sized samples. This study was limited by the unbalanced sizes of the groups, especially the smaller size of the S group, which limited the confidence of the reported results (a difference of one person in the classification would mean a gain or loss of 5% in sensitivity). This is a consequence of the difficulty of spotting suspects of simulation in a realistic setting.

## Data Availability

Data are available upon reasonable request to the authors.

## Annex A

**Table 5:** Neck Pain Symptoms Questionnaire (NPSQ)

**Instructions:** *The following questionnaire contains a series of statements describing typical symptoms of people suffering from neck pain. When you agree with a statement, or when you believe that it is true or usually true for you, check the box TRUE. When you do not agree with a statement, or when you believe that it is false or usually false for you, check the box FALSE. Please, DON’T SKIP ANY STATEMENT, and respond to all the statements to the best of your ability, even if some of them are difficult or seem inappropriate for you. Before responding to the statements, please insert your name, your gender, your age, your date of birth, and today’s date in the appropriate spaces*		
1	My neck pain increases gradually during the day	□TRUE □FALSE
2	My neck pain is continuously present all day	□TRUE □FALSE
3	My neck pain is variable during the day	□TRUE □FALSE
4	My neck pain spreads to the head area	□TRUE □FALSE
5	My neck pain spreads to the shoulders and/or hands	□TRUE □FALSE
6	I often suffer from muscle stiffness	□TRUE □FALSE
7	Sometimes I feel pins and needles or numbness in the arms	□TRUE □FALSE
8	I often suffer from headaches	□TRUE □FALSE
9	I experience sleep disturbances	□TRUE □FALSE
10	My neck pain comes along with feelings of dizziness and/or some nausea	□TRUE □FALSE
11	I have started to experience mood swings	□TRUE □FALSE
12	My neck pain gets worse when reading and watching television	□TRUE □FALSE
13	My neck pain gets worse when working and/or doing housework	□TRUE □FALSE
14	My neck pain gets worse when carrying heavy or medium objects	□TRUE □FALSE
15	My neck pain gets worse when driving	□TRUE □FALSE
16	My neck pain gets worse when lifting weights	□TRUE □FALSE
17	My neck pain gets worse with prolonged postures	□TRUE □FALSE
18	My neck pain gets worse due to stress	□TRUE □FALSE
19	My neck pain gets worse with awkward postures	□TRUE □FALSE
20	My neck pain gets worse when completing most self-care actions	□TRUE □FALSE
21	I find it difficult to turn my neck quickly	□TRUE □FALSE
22	My neck pain is widespread outside of the cervical and upper thoracic region	□TRUE □FALSE
23	My neck pain is not relieved by any medication	□TRUE □FALSE
24	My neck pain is not relieved by anything	□TRUE □FALSE
25	I think that my neck pain will never be relieved	□TRUE □FALSE
26	My neck pain always forces me to stay in bed	□TRUE □FALSE
27	Sometimes my neck pain prevents me from walking	□TRUE □FALSE
28	I lost sensibility in both upper limbs	□TRUE □FALSE
29	I lost strength in both upper limbs	□TRUE □FALSE
30	I feel that I lost strength in whole body	□TRUE □FALSE
31	I lost coordination in my upper limbs and/or hands	□TRUE □FALSE
32	I struggle to manipulate objects with my hands	□TRUE □FALSE
33	My neck pain gets worse with light touch and/or light pinching of the skin	□TRUE □FALSE
34	My neck pain gets worse with slow and controlled movements	□TRUE □FALSE
35	My neck pain gets worse with minor temperature and/or humidity changes	□TRUE □FALSE
36	I totally lost my memory and concentration ability	□TRUE □FALSE
37	I have started to experience hand tremors	□TRUE □FALSE
38	I have started to have vision problems	□TRUE □FALSE
39	I have started to have difficulty swallowing	□TRUE □FALSE
40	I noticed alterations of the colour and roughness of the skin in the neck area	□TRUE □FALSE
41	My neck pain gets worse when eating specific food	□TRUE □FALSE
42	Sometimes I hear a constant sound in my ears	□TRUE □FALSE
43	It happened that my neck pain has given me hallucinations	□TRUE □FALSE
44	It happened that my neck pain has prevented me from recognizing people, places, or situations that are generally familiar to me	□TRUE □FALSE
45	Once a week, suddenly I get cold despite it being very hot outside	□TRUE □FALSE
46	I happened to look at myself as if I were outside of my body	□TRUE □FALSE
47	My neck pain comes along with an inconvenience in my physical appearance that I consider intolerable to other people	□TRUE □FALSE
48	When my neck hurts, sometimes I feel emotionally “anesthetized”	□TRUE □FALSE
49	When my neck hurts, sometimes I hear voices in my head	□TRUE □FALSE
50	Recently, I perceive involuntary movements in the muscles of the face that I cannot control	□TRUE □FALSE
51	When my neck hurts a lot, I am not able to count from 1 to 10	□TRUE □FALSE
52	Recently, I have not noticed any changes in my sense of smell	□TRUE □FALSE
53	It seems that food no longer has the same taste it had once	□TRUE □FALSE
54	I happened to feel like some larvae were walking under the skin of my neck	□TRUE □FALSE
55	Sometimes my neck pain prevents me from talking	□TRUE □FALSE
56	When my neck hurts, I find it difficult to understand what people are telling me	□TRUE □FALSE
57	Even when my neck hurts, I can converse with other people	□TRUE □FALSE
58	My neck pain has not affected my memory	□TRUE □FALSE
59	I have not noticed the appearance of bruises in the neck area	□TRUE □FALSE
60	My neck pain does not prevent me from properly drinking from a glass	□TRUE □FALSE
61	I have never lost consciousness because of severe neck pain	□TRUE □FALSE
62	Recently, I have not noticed an unusual difficulty in reaching orgasm	□TRUE □FALSE
63	I never felt a strong heat around my neck, like it was burning	□TRUE □FALSE
64	My neck pain doesn’t come with sudden tachycardia	□TRUE □FALSE
65	I have no difficulty breathing and/or I don’t often experience shortness of breath	□TRUE □FALSE

Possible symptoms: questions 1 to 21

Rare symptoms: questions 22 to 35

Impossible symptoms: questions 26 to 65

Scoring: TRUE = 1 FALSE = 0

(the score of questions 52, 57–65 must be reversed)

## Code availability

Data analysis code is available upon reasonable request to the authors.
